# The Pullback Pressure Gradient: Transforming Invasive Coronary Physiology from Lesion Assessment to Disease Pattern Characterization—A Perspective

**DOI:** 10.3390/medicina61112034

**Published:** 2025-11-14

**Authors:** Artur Dziewierz, Barbara Zdzierak, Stanisław Bartuś, Wojciech Zasada

**Affiliations:** 1 2nd Department of Cardiology, Institute of Cardiology, Jagiellonian University Medical College, 30-688 Krakow, Poland; barbarazdzierak@gmail.com (B.Z.); mbbartus@cyfronet.pl (S.B.); 2 Clinical Department of Cardiology and Cardiovascular Interventions, University Hospital, 30-688 Krakow, Poland; zasada.wojciech@gmail.com; 3 KCRI, 30-347 Krakow, Poland

**Keywords:** pullback pressure gradient, coronary physiology, fractional flow reserve, instantaneous wave-free ratio, focal coronary disease, diffuse coronary disease, outcomes

## Abstract

This perspective comprehensively analyzes the invasive pullback pressure gradient (PPG), a novel physiological index that characterizes the longitudinal distribution of coronary artery disease and guides revascularization strategy modified in 14% of patients in the PPG Global Registry based on PPG assessment. We trace the historical development from subjective pullback curve analysis to a standardized, quantitative metric and describe the procedural aspects of both motorized and manual PPG acquisition. We synthesize evidence supporting PPG’s clinical utility in predicting post-percutaneous coronary intervention outcomes, guiding revascularization decisions, and improving patient-centered outcomes. A central focus is PPG’s mechanistic role in explaining the physiological basis of discordance between fractional flow reserve (FFR) and instantaneous wave-free ratio (iFR), linking focal disease to FFR-positive/iFR-negative patterns and diffuse disease to FFR-negative/iFR-positive patterns. We conclude that PPG represents a fundamental advancement in coronary physiology, shifting clinical focus from individual stenoses to overall disease patterns. This paradigm shift provides deeper understanding of coronary artery disease pathophysiology and offers a powerful predictive tool to guide personalized revascularization strategies. Prospective randomized trials will be essential to solidify its role as a cornerstone of modern interventional cardiology practice.

## 1. Introduction

### 1.1. The Limitations of Angiography and the Dawn of Coronary Physiology

For decades, the diagnosis and management of coronary artery disease (CAD) relied on visual interpretation of the coronary angiogram [1,2]. While invaluable as an anatomical roadmap, this technique provides only a two-dimensional silhouette of the coronary lumen—a “luminogram”—that repeatedly underestimates the true burden and distribution of atherosclerosis [2,3,4]. Reliance on angiography alone often leads to misjudgment of both lesion severity and overall disease pattern, creating a substantial gap between anatomical appearance and functional significance [4,5].

The paradigm shifted in the 1990s with introduction of the intracoronary pressure wire and validation of fractional flow reserve (FFR) [2,5,6]. FFR is the ratio of mean pressure distal to a stenosis (Pd) to mean aortic pressure (Pa) during maximal, steady-state hyperemia [6,7]. It represents maximal achievable blood flow in a stenotic artery as a fraction of what would occur in the hypothetical absence of stenosis. Landmark trials demonstrated that FFR-guided percutaneous coronary intervention (PCI) was superior to angiography-guided PCI, improving clinical outcomes and cost-effectiveness [2,8]. Consequently, FFR became the gold standard for assessing hemodynamic significance of intermediate-grade coronary stenoses and earned strong recommendations in clinical practice guidelines [6,8,9,10]. This evolution marked a critical transition from purely anatomical assessment of CAD to functional evaluation, shifting the clinical question from “Is there a stenosis?” to “Does the stenosis cause ischemia?”

### 1.2. The Unanswered Question: FFR as a “Spot” Measurement

Despite its revolutionary impact, standard FFR provides only a single, distal pressure measurement. This “spot” FFR value represents cumulative pressure loss along the entire epicardial vessel up to the measurement point [4,11]. A positive FFR (typically ≤0.80) confirms ischemia but does not delineate how that pressure loss is distributed. It cannot distinguish whether the ischemic burden stems from a single focal stenosis, a long segment of diffuse atherosclerosis, or a combination of both [1,2,4,7]. This distinction carries profound clinical importance. The underlying pathophysiological disease pattern has significant therapeutic and prognostic implications. Diffuse CAD is associated with persistent angina after PCI, lower post-procedural FFR values, and higher incidence of future adverse cardiovascular events [12,13]. Treating diffuse disease with a focal stent may fail to restore normal vessel conductance and yield suboptimal clinical results.

The pressure pullback maneuver—slow withdrawal of the pressure wire from distal vessel to coronary ostium while continuously recording pressure—was initially recommended simply to check for signal drift [7,11]. However, operators recognized that the resulting pressure-versus-length curve offered qualitative insight into the spatial distribution of pressure losses. Sharp “step-ups” in the FFR curve visually correlated with focal lesions, while gradual, steady declines suggested diffuse disease [4,14]. Despite its conceptual appeal, this visual interpretation was highly subjective, lacked standardization, and suffered from significant inter-observer variability [14]. These limitations prevented widespread adoption as a reliable diagnostic tool and highlighted the need for an objective, quantitative method to analyze pullback curves and characterize disease patterns.

### 1.3. Invasive Pullback Pressure Gradient

Invasive pullback pressure gradient (PPG) is an intracoronary hemodynamic measurement derived from pressure wire pullback within a coronary artery [1,2,4,7,12,15,16,17]. It quantitatively assesses the longitudinal distribution of epicardial resistance, characterizing atherosclerotic disease patterns as focal or diffuse. PPG is an invasive procedure performed in the cardiac catheterization laboratory and should be distinguished from photoplethysmography, which shares the same abbreviation in medical literature.

## 2. The Pullback Pressure Gradient (PPG): From Physiological Concept to Clinical Calculation

### 2.1. Conceptual Development: Standardizing the Pullback Curve

The conceptual leap from qualitative visual assessment of the FFR pullback curve to a quantitative index established PPG as a clinical tool. The goal was to overcome subjectivity and poor reproducibility of visual interpretation by creating a standardized, objective numeric metric that reliably characterizes coronary disease patterns [4,7,12,15].

The PPG index quantifies information contained within the entire pullback curve, integrating both the magnitude of pressure drops and their spatial distribution along the vessel. This approach transforms the visual curve pattern into a single continuous metric ranging from 0 to 1. Values approaching 1 indicate predominantly focal disease, characterized by a large, abrupt pressure drop over a short segment. Conversely, values approaching 0 signify predominantly diffuse disease, characterized by gradual, continuous re loss along a significant vessel length [4,7,12,15,16]. This provides standardized language to describe the pathophysiological spectrum of CAD (Table 1).

### 2.2. The Invasive Procedure and Calculation of the PPG Index

PPG data acquisition extends the standard FFR procedure performed in the cardiac catheterization laboratory.

- Initial setup and wire positioning: The procedure begins with standard coronary angiography and engagement of the target vessel with a guiding catheter. A 0.014-inch pressure-sensing guidewire (e.g., PressureWire X, Abbott Vascular) is prepared, calibrated, and advanced through the guiding catheter. Pressure from the guidewire sensor is “equalized” with pressure from the guiding catheter tip to ensure identical readings in the absence of a gradient [4,7,12,15]. The pressure wire is then advanced until its sensor is positioned in the distal third of the coronary artery, typically in a segment with a visually estimated diameter of at least 2.0 mm [4,7,12,15].
- Inducing hyperemia: To measure hyperemic PPG, maximal and stable coronary vasodilation must be achieved. This is most commonly accomplished via continuous intravenous adenosine infusion at 140 μg/kg/min for at least 2 min to ensure a steady-state hyperemic plateau [4,7,12,15].
- The pullback maneuver: Once stable hyperemia is established, the pressure wire is withdrawn slowly at constant speed from its distal position to the guiding catheter tip while continuously recording pressure from both wire and catheter [4,7,12,15]. Two techniques are available:
  *Motorized pullback:* This method, used in initial validation studies, ensures perfectly constant and known pullback speed (typically 1 mm/s) using a dedicated motorized device [7,15]. This technique provided rigorous standardization of the pressure-length relationship necessary for index development and validation [7,15]. However, these devices are not widely available and can be cumbersome, representing a barrier to routine clinical use.
  *Manual pullback:* This is the current standard clinical technique, whereby the operator manually withdraws the wire over 20 to 30 s [4,7,12,15]. Translation of PPG from research concept to practical clinical tool critically depended on demonstrating that manual pullbacks yield accurate and reproducible results. A key validation study demonstrated excellent agreement between manual and motorized pullbacks (mean difference −0.01 ± 0.07) under controlled laboratory conditions. However, translation to routine clinical practice remains operator-dependent and requires adequate training to maintain the slow, steady pullback technique essential for achieving comparable results. The laboratory validation provides necessary—but not sufficient—evidence for clinical implementation [4,7,12,14,15].
- Calculation of the PPG index: The PPG index is computed from two primary components derived from the hyperemic FFR pullback curve: (1) the magnitude of the largest localized pressure gradient, and (2) the overall extent of functional disease along the vessel [4,7,12,15].

*Formula for motorized pullback:* The original distance-based formula is:

$${\mathrm{PPG}}_{\mathrm{index}}={\left\{\frac{{\mathrm{MaxPPG}}_{20\mathrm{mm}}}{\Delta {\mathrm{FFR}}_{\mathrm{vessel}}}+(1-\frac{{\mathrm{Length}}_{\mathrm{functional}\;\mathrm{disease}}}{{\mathrm{Length}}_{\mathrm{total}\;\mathrm{vessel}}})\right\}}^{2}$$

Here, MaxPPG_20mm_ represents the maximum FFR change observed over any 20 mm vessel segment, quantifying the magnitude of the most focal pressure drop [7,15]. ΔFFRᵥₑₛₛₑₗ is the total pressure loss along the entire vessel from ostium to distal measurement point [7,15]. The Length functional disease is the cumulative vessel length (in millimeters) where the FFR drop equals or exceeds a predefined threshold of 0.0015/mm, quantifying the spatial extent of disease [7,15].

*Formula for manual pullback:* To accommodate variability in speed and length of manual pullback, the formula was adapted to be time-based rather than distance-based. The key modification defines the maximal pressure gradient as the largest FFR change occurring over 20% of total pullback duration [7,15]. This adaptation makes the calculation robust and independent of the precise withdrawal speed, a crucial factor for clinical utility.

### 2.3. Emerging Techniques: Non-Hyperemic Pullback Pressure Gradient

Coronary physiology has consistently evolved toward simplification and elimination of hyperemic agents, which add procedural time, cost, and potential adverse effects. Development of iFR as an alternative to FFR exemplified this trend. Following this trajectory, research now explores the utility of PPG derived from pressure pullback performed under resting (non-hyperemic) conditions [18].

**Figure 1:**
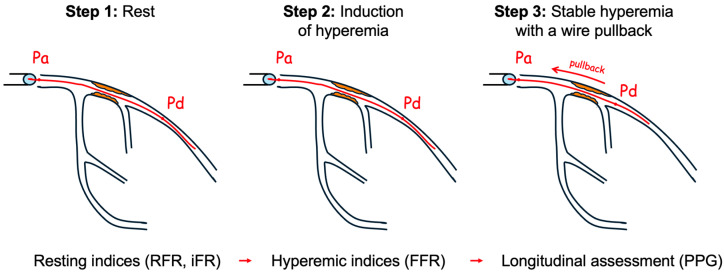
Sequential steps in invasive coronary physiology assessment. Step 1: At rest, the pressure wire measures aortic pressure (Pa) and distal coronary pressure (Pd) to calculate resting indices (RFR, iFR). Step 2: Following adenosine administration to induce maximal hyperemia, the same pressures are recorded to calculate fractional flow reserve (FFR). Step 3: During stable hyperemia, the pressure wire is slowly withdrawn from the distal vessel toward the guide catheter, generating a continuous Pa-Pd pressure gradient along the vessel length. This pullback trace enables calculation of the pullback pressure gradient (PPG), which quantifies the longitudinal distribution and functional significance of coronary disease.

Initial studies are promising. A prespecified sub-analysis of the PPG Global Registry directly compared resting PPG to reference-standard hyperemic PPG in patients with hemodynamically significant lesions, demonstrating strong correlation (r = 0.80) and substantial agreement (Cohen κ = 0.64) between methods—Table 2 [18]. Furthermore, resting PPG showed excellent capacity to predict optimal post-PCI FFR (AUC: 0.76). These findings suggest that non-hyperemic pullback may provide a reliable, streamlined method for quantifying CAD patterns, potentially broadening the application of longitudinal physiological assessment in the catheterization laboratory (Figure 1).

## 3. Clinical Applications: PPG as a Predictive and Procedural Guidance Tool

The clinical applications of PPG extend beyond anatomic disease classification. PPG provides quantitative guidance at multiple decision points: pre-procedurally for selecting revascularization strategy, intra-procedurally for optimizing intervention execution, and post-procedurally for assessing residual ischemia and predicting outcomes.

### 3.1. Predicting the Functional Outcome of PCI

PPG predicts the functional result of PCI before stent deployment [4,7,12,15,16]. While low distal FFR identifies vessels that may benefit from revascularization, it cannot quantify the pressure gradient recovery achievable by focal stenting. PPG addresses this gap.

The PPG Global study evaluated pre-procedural PPG’s capacity to predict post-PCI FFR ≥ 0.80 in a large-scale prospective international registry [4,12]. Results were striking: pre-PCI PPG strongly correlated with FFR change after the procedure (r = 0.65) and demonstrated excellent capacity to predict optimal revascularization, with an area under the receiver operating characteristic curve (AUC) of 0.82 [12]. In contrast, baseline distal FFR alone had no predictive power for post-PCI outcome (AUC 0.54).

High PPG values concentrate pressure loss in discrete segments, making focal intervention highly effective [4,7,12,15]. Conversely, low PPG values indicate distributed pressure loss along the vessel, where focal stenting leaves substantial residual disease untreated, resulting in suboptimal post-PCI FFR [13,20,21,22].

### 3.2. Guiding Revascularization Strategy and Procedural Planning

**Figure 2:**
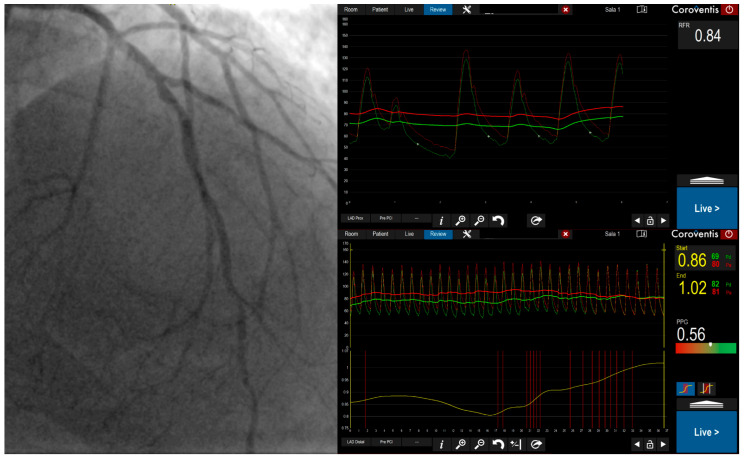
Invasive Coronary Angiography with Physiological Assessment Demonstrating Diffuse Coronary Artery Disease. A 78-year-old male with chronic heart failure with reduced ejection fraction, hypertension, and hypercholesterolemia underwent invasive coronary angiography with physiological assessment of the LAD. Angiography revealed diffuse atherosclerotic changes throughout the vessel. RFR was 0.83 and FFR during maximal hyperemia was 0.86. RFR met the ischemic threshold (≤0.89), while FFR did not (≤0.80), demonstrating FFR-negative/RFR-positive discordance. PPG was 0.56, confirming predominantly diffuse disease pattern. This physiological profile-positive resting index with negative hyperemic index and low PPG-is characteristic of diffuse coronary artery disease driven primarily by cumulative frictional losses along the vessel length. Given the diffuse disease pattern and unfavorable predicted response to PCI, the patient was managed with optimal medical therapy.

By providing a second, independent dimension of physiological information-disease pattern-PPG fundamentally enhances clinical decision-making [4,7,12,15]. Assessment evolves from a binary FFR cutoff-based choice to nuanced judgment incorporating disease distribution and likelihood of successful intervention. Evidence from the PPG Global study demonstrates this impact in real-world practice. Incorporating PPG analysis into decision-making led to revascularization strategy changes in 14% of patients after pullback reviews [12]. In these cases, PPG information redirected patients from PCI toward more appropriate alternative treatments, such as coronary artery bypass grafting (CABG) for complex diffuse disease, or optimal medical therapy (OMT) when significant functional gain from PCI was deemed unlikely [4,7,12,15,23]. Treatment strategy changes occurred in approximately 25% of cases overall and significantly more frequently in patients with diffuse disease (low PPG), where the limitations of focal stenting are most evident (Figure 2).

Even when PCI is the chosen strategy, the PPG pullback curve serves as a physiological roadmap to guide and optimize the procedure. In the same registry, operators modified stent length decisions in 22.1% of cases after reviewing pullback data [12]. This enables more precise therapy application, ensuring stents cover segments responsible for the largest pressure drops while avoiding unnecessary stenting of hemodynamically insignificant areas. This physiology-guided approach to stent selection associated with better outcomes; suboptimal post-PCI FFR occurred more frequently when PCI strategy was not adapted based on PPG information (55.5% vs. 45.6%) [12].

### 3.3. Correlation with Patient-Centered Outcomes and Procedural Safety

PPG’s clinical utility extends to physiological endpoints. Growing evidence links pre-procedural disease pattern, as quantified by PPG, to both patient-reported outcomes and intervention safety. Patients with high pre-PCI PPG values (focal disease) report significantly greater improvement in angina symptoms and overall quality of life following PCI [12]. Conversely, residual angina after angiographically successful PCI is a common clinical problem, and diffuse atherosclerosis is one of its primary causes. Residual angina following PCI occurs nearly twice as frequently in patients with low PPG compared to high PPG (51.9% vs. 27.5%; *p* = 0.020), suggesting that diffuse disease predicts incomplete symptom relief despite angiographically successful revascularization [4,7,12,15,23]. This finding helps manage patient expectations and provides physiological explanation for persistent symptoms despite technically successful intervention.

Furthermore, PPG provides crucial information regarding procedural safety. Treatment of diffuse disease often requires more complex procedures, including more and longer stents, which increases the risk of complications such as side branch occlusion, stent-edge dissection, and periprocedural myocardial infarction [4,7,12,15,23]. The PPG Global study confirmed this association, finding that periprocedural myocardial infarction occurred significantly more frequently in patients with low PPG values (<0.62) compared to those with focal disease (odds ratio 1.71) [12]. This observation suggests a link between pre-procedural physiological pattern and acute procedural risk, adding another critical variable to the risk-benefit calculation for PCI.

**Figure 3:**
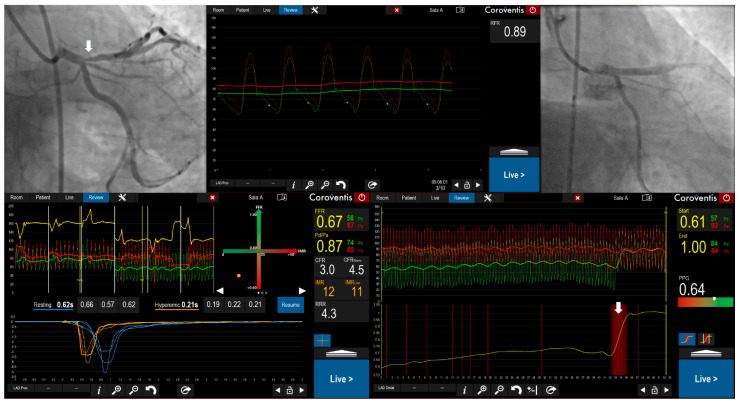
Invasive Coronary Angiography with Physiological Assessment Demonstrating Focal Coronary Artery Disease. A 56-year-old male with coronary artery disease (prior PCI to the mid-LAD), hypertension, hypercholesterolemia, and persistent CCS class II angina underwent invasive coronary angiography with physiological assessment of LAD. Angiography revealed 60% stenosis in the proximal LAD (white arrow). Resting full-cycle ratio (RFR) was 0.89 and fractional flow reserve (FFR) during maximal hyperemia was 0.67. Both values met ischemic thresholds (RFR ≤ 0.89, FFR ≤ 0.80), confirming hemodynamically significant epicardial stenosis. Pullback pressure gradient (PPG) was 0.64, indicating an intermediate disease pattern with mixed focal and diffuse characteristics, though with tendency toward focal disease in the proximal segment (change in the pressure gradient related to the proximal LAD stenosis marked with white arrow). The patient underwent successful PCI to the proximal LAD and left main coronary artery.

The concept of physiology-guided incremental optimization strategy, where additional interventions improve suboptimal post-PCI FFR, is also influenced by underlying disease pattern. One study found that physiology-guided incremental optimization strategy was applied more frequently to vessels with diffuse disease. Yet despite these additional efforts, patients who started with focal disease still achieved significantly higher final post-PCI FFR values, underscoring the inherent difficulty of achieving complete functional revascularization in diffuse CAD (Figure 3).

## 4. Resolving a Clinical Conundrum: The Role of PPG in FFR-iFR Discordance

### 4.1. The Clinical Problem: FFR-iFR Discordance

Introduction of non-hyperemic pressure ratios, notably iFR and resting full-cycle ratio, advanced clinical practice by simplifying physiological assessment [3,14,17,24,25,26,27]. By eliminating adenosine, these indices made physiological testing faster, less expensive, and more comfortable for patients. Large-scale randomized trials demonstrated that iFR-guided revascularization was non-inferior to the gold-standard FFR-guided approach, cementing its place in clinical guidelines [2,8]. However, a persistent challenge emerged: in approximately 20% of cases, FFR and iFR provide discordant results [3,14,17,24,25,26,27]. For the same coronary stenosis, one index suggests hemodynamic significance while the other does not. Standard cutoffs for significance are FFR ≤ 0.80 and iFR ≤ 0.89 [8]. This discordance creates significant clinical uncertainty, leaving operators questioning which index to trust and how to proceed with treatment. While numerous clinical factors associate with discordance—including patient age, sex, diabetes mellitus, chronic kidney disease, heart rate, lesion location (e.g., proximal left anterior descending artery), and microvascular dysfunction—these associations did not provide a unifying physiological mechanism to explain the phenomenon [3,14,17,24,25,26,27,28,29,30]. Recent multicenter analyses confirmed that PPG quantitatively accounts for most FFR–iFR discordance [12,15,23]. Specifically, focal disease (high PPG) correlates with FFR-positive/iFR-negative discordance, whereas diffuse disease (low PPG) associates with the converse pattern (FFR-negative/iFR-positive).

### 4.2. The Mechanistic Link: Disease Pattern as the Primary Determinant of Discordance

Development of PPG and its ability to objectively quantify disease pattern has provided the crucial missing piece to the discordance puzzle. Compelling evidence from multiple independent studies now firmly establishes that physiological disease pattern is a primary determinant of FFR/iFR discordance type [3,14,17,24,25,26,27]. The relationship is not random but follows a clear, predictable pattern, transforming discordance from a frustrating disagreement between two tests into a source of deeper diagnostic information about underlying pathophysiology.

- FFR-positive/iFR-negative (FFR+/iFR−) discordance: This pattern, where the lesion is deemed significant by the hyperemic index (FFR) but not by the resting index (iFR), strongly and consistently associates with physiologically focal disease, indicated by high PPG [17]. In a recent multicenter study, 76.3% of vessels with FFR+/iFR− discordance demonstrated predominantly focal disease pattern [17]. Another study found 58.5% of FFR+/iFR− lesions were focal [14].
- FFR-negative/iFR-positive (FFR−/iFR+) discordance: This pattern, where the lesion is non-significant during hyperemia (FFR) but significant at rest (iFR), equally strongly associates with physiologically diffuse disease, indicated by low PPG [17]. The same multicenter study found that 96.3% of vessels with FFR−/iFR+ discordance exhibited predominantly diffuse disease pattern [17]. Another study found 81.6% of FFR−/iFR+ lesions were diffuse [14]. Median PPG was significantly lower in the FFR−/iFR+ group compared to the FFR+/iFR− group (0.65 vs. 0.82) [26].

### 4.3. The Underlying Hemodynamics: Frictional Loss Versus Separation Loss

This consistent link between disease pattern and discordance type is not mere correlation; it is explained by fundamental fluid dynamics principles governing pressure loss in stenotic vessels [14,31]. Total pressure drop (Δ*P*) across a coronary lesion comprises two primary energy loss components:

1. Frictional loss ($\Delta P_{f}$): Energy lost due to viscous friction between flowing blood and the endothelial vessel wall surface. This loss is linearly proportional to flow velocity (*v*) and stenotic segment length (*L*), representing the dominant pressure loss mode in long, diffusely diseased arteries:

$$\Delta P_{f}\propto v\cdot L$$

2. Separation loss ($\Delta P_{s}$): Energy lost due to turbulence, eddy currents, and flow separation occurring at the abrupt exit of a discrete focal stenosis. This loss is proportional to the square of flow velocity (*v*^2^). Because of this squared relationship, separation loss is minimal at low (resting) flow rates but becomes exponentially larger as flow accelerates during hyperemia:

$$\Delta P_{s}\propto v^{2}$$

These physical principles provide clear mechanistic explanation for observed discordance patterns (Table 3):

- Explaining FFR+/iFR− in focal disease: A classic focal stenosis is short in length but severely narrowed. At rest, blood flow velocity is relatively low. Frictional loss is minimal because the lesion is short, and separation loss is minimal because flow velocity is not yet squared. Therefore, total pressure drop is small, resulting in negative iFR (iFR > 0.89). However, during hyperemia, adenosine administration dramatically increases flow velocity (often 2–3 times resting rate). This velocity surge profoundly affects the separation loss component due to the *v*^2^ term, causing large pressure drop across the stenosis and resulting in positive FFR (FFR ≤ 0.80) [14,31].
- Explaining FFR−/iFR+ in diffuse disease: The FFR−/iFR+ discordance pattern reflects a combination of epicardial and microvascular factors. In long, diffusely diseased vessels, high cumulative frictional loss dominates hemodynamics, causing significant pressure drop even at rest and resulting in a positive iFR. However, these patients frequently harbor coexisting microvascular dysfunction, particularly in those with advanced age and diabetes [14,31,32]. This microvascular impairment is quantitatively captured by elevated index of microcirculatory resistance (IMR) or reduced coronary flow reserve (CFR). Microvascular dysfunction fundamentally alters the vessel’s response to hyperemia. The high-resistance distal bed impairs microcirculatory vasodilation in response to adenosine, creating a “physiological cap” on achievable hyperemic flow velocity. This flow limitation has critical hemodynamic consequences: because hyperemic pressure losses—both frictional and separation—are flow-dependent, blunted flow velocity directly attenuates the total pressure gradient. In diffusely diseased vessels, this attenuated flow results in a smaller-than-expected pressure drop across the epicardial disease, often insufficient to push FFR below its ischemic threshold of 0.80 despite substantial epicardial atherosclerotic burden. Therefore, the FFR−/iFR+ discordant pattern (low PPG) serves as a powerful diagnostic signature not merely of diffuse epicardial disease, but of global coronary vasculopathy encompassing both diffuse epicardial atherosclerosis and compromised microcirculatory function (high IMR) [14,31,32].

### 4.4. Clinical Implications of Understanding Discordance

The insight provided by PPG resolves the clinical dilemma of FFR/iFR discordance by serving as a physiological “tie-breaker” that reveals the underlying disease pattern driving disparate results [33].

- FFR+/iFR− discordance (focal disease signature): This pattern indicates a lesion that is an ideal PCI target. Positive FFR confirms significant ischemia under stress, and the focal disease nature (high PPG) predicts that stenting will abolish separation losses, yielding substantial functional gain and favorable clinical outcome [14,17,26,31].
- FFR−/iFR+ discordance (diffuse disease signature): This pattern suggests a more challenging clinical scenario. Positive iFR indicates significant resting pressure loss, but the diffuse disease nature (low PPG) predicts that focal stenting will provide only modest functional improvement, likely resulting in suboptimal post-PCI FFR and higher probability of residual symptoms [14,17,26,31]. In this scenario, clinicians may favor optimal medical therapy or CABG (Figure 3).

While observational studies suggest that clinical outcomes of medically managed discordant lesions are generally favorable and similar to concordantly negative lesions [14,15,17,31], this does not diminish the value of mechanistic understanding. PPG provides a rational, physiological basis for decision-making tailored to individual patient disease patterns, rather than relying solely on discordant numerical values.

## 5. Conclusions and Future Horizons

### 5.1. Summary of Evidence and Integration into Clinical Practice for the Interventional Cardiologist

PPG has matured from a research concept into a clinically validated tool that provides indispensable information for the contemporary interventional cardiologist. Evidence strongly supports its integration into practice as a complement to, not replacement for, FFR and iFR. While FFR/iFR determine whether ischemia is present, PPG answers the critical follow-up questions of how disease is distributed and whether PCI is likely to be effective and safe.

- Pre-PCI planning and strategy: Routine pressure pullback to calculate PPG may be considered in all patients with hemodynamically significant lesions (e.g., FFR ≤ 0.80) being considered for PCI. PPG should be used to phenotype disease and forecast procedural outcome. High PPG (e.g., >0.75) strongly supports PCI, predicting favorable functional results and greater symptom relief [12,14,15,19,21,23]. Conversely, low PPG (e.g., <0.62) identifies patients with diffuse disease, in whom PCI benefit is less certain and procedural risk is higher [12,14,15,19,21,23]. This should prompt careful reconsideration of the risk-benefit ratio and discussion of alternative strategies such as CABG or OMT, particularly in patients with stable angina.
- Intra-PCI guidance: The pullback curve serves as a physiological angiogram. It should be used to precisely plan stent length and placement, ensuring segments responsible for the largest pressure gradients are fully covered, thereby maximizing procedural functional gain [12,14,15,19,21,23,34].
- Interpreting discordance: In cases of FFR/iFR discordance, performing pullback to determine PPG can resolve clinical uncertainty. As outlined in Section 4, the disease pattern revealed by PPG provides clear physiological rationale for discordance and guides appropriate therapeutic strategy [12,14,15,17,19,21,23,26].

### 5.2. Key Messages for the General Cardiologist and General Practitioner

Insights from PPG have important implications extending beyond the catheterization laboratory. For non-invasive cardiologists and primary care physicians managing patients with CAD long term, understanding disease patterns is crucial for patient counseling and interpreting clinical course.

- Managing patient expectations: A simple analogy explains the difference between focal and diffuse disease. Focal blockage resembles a single large boulder in a river; removing it with a stent effectively restores flow. In contrast, diffuse disease resembles a long, shallow, rocky river stretch; placing a short paved section (a stent) may not significantly improve overall flow [15]. This helps patients understand why stenting may not be a complete solution for everyone.
- Understanding treatment decisions: This framework provides clear rationale for why interventional cardiologists might recommend medical therapy or CABG even when physiological tests like FFR or iFR are “positive.” Decisions are based not solely on ischemia presence but on sophisticated assessment of disease pattern and predicted durability and effectiveness of PCI.
- Interpreting post-PCI symptoms: Persistent angina after technically successful PCI is a common clinical challenge. Knowledge that diffuse disease (identified by low pre-PCI PPG) is a primary cause of this phenomenon is invaluable [15]. This allows physicians to reassure patients that their symptoms are not necessarily indicative of procedural failure or acute stent complications but rather reflect the underlying chronic, diffuse atherosclerosis that was not fully correctable with focal stenting. This understanding guides appropriate medical management and prevents unnecessary repeat diagnostic procedures [34].

### 5.3. Limitations and Unanswered Questions

Despite its proven utility, current limitations of PPG and areas requiring further investigation must be acknowledged.

- Technical considerations: Although manual pullback has been validated as highly reproducible, it is a technique-dependent maneuver requiring operators to withdraw the wire at slow, steady pace to avoid artifacts. Sudden movements or wire whip can distort pressure tracings [12,15,18,19,21,23,26]. Standard hyperemic PPG also requires adenosine administration, a limitation that resting PPG development aims to overcome.
- Lack of a universal cutoff value and the importance of the continuous spectrum: A major limitation is the absence of a single, universally accepted PPG value to dichotomously classify disease. The literature reflects significant heterogeneity in thresholds. The PPG Global study used a median value of <0.62 to define higher periprocedural myocardial infarction risk [12], while FFR-iFR discordance studies have employed <0.75 to define diffuse disease [26], and other analyses have used >0.73 to identify focal patterns [14]. This variability highlights that cutoffs are often derived post hoc from specific study populations and may lack generalizability. Therefore, PPG must be interpreted as a continuous variable rather than a binary test. The clinical principle is straightforward: the lower the PPG, the more diffuse the disease and the greater the caution required before proceeding with PCI. This spectrum also reveals a substantial “gray zone” of intermediate disease patterns (PPG 0.60–0.75), as exemplified in Figure 3 (PPG 0.64), which represent mixed pathology. These cases are likely suboptimal PCI targets but may not warrant outright deferral, representing a key area for future research into nuanced risk-benefit assessment and shared decision-making [15].
- Evidence gaps/external validation: Despite robust findings from the PPG Global Registry—including an AUC of 0.82 for predicting optimal revascularization and strong associations with post-PCI functional outcomes—independent prospective validation studies remain essential. Until such data is available, the generalizability of this predictive value remains a key unanswered question. Furthermore, the majority of PPG research has been conducted in patients with single-vessel disease already considered PCI candidates. Its applicability and predictive value in more complex patient subsets—such as those with diffuse multivessel disease, left main disease, or acute coronary syndromes—require further dedicated investigation [12,15,16,18,19,21,23,26].

### 5.4. Future Research Directions: From Prediction to Prescription

Current evidence has firmly established PPG as a powerful diagnostic and predictive tool. The next research frontier must focus on translating this predictive capacity into a prescriptive one, demonstrating through rigorous trials that PPG-guided strategy leads to superior clinical outcomes.

- The definitive randomized controlled trial: The most critical unmet need is a large-scale randomized controlled trial stratifying patients with hemodynamically significant ischemia (FFR ≤ 0.80) by PPG. Patients with low PPG (diffuse disease) could be randomized to optimal medical therapy versus CABG versus optimized PCI with long stents to determine the best management strategy for diffuse atherosclerosis. Patients with high PPG (focal disease) could be randomized to focal PCI versus optimal medical therapy to definitively test whether revascularization improves clinical outcomes in this theoretically ideal subgroup. Primary endpoints should include a composite of long-term major adverse cardiovascular events and patient-reported outcomes such as angina relief and quality of life. For patients with low PPG and significant ischemia, in whom PCI is predicted to be suboptimal and CABG may not be an option, OMT is the current standard. However, this leaves a therapeutic gap for those with refractory angina. Future research should investigate novel device-based therapies for this specific phenotype. The coronary sinus reducer is a promising percutaneous device designed for patients with refractory angina who are not candidates for conventional revascularization [35,36]. A future trial randomizing patients with low FFR and low PPG to either OMT alone or OMT plus coronary sinus reducer implantation would be a critical step in establishing a new, targeted treatment pathway for diffuse, non-revascularizable disease.
- Defining optimal therapy for diffuse disease: A key randomized controlled trial question is the optimal management strategy for patients with physiologically significant ischemia (low FFR) but diffuse disease pattern (low PPG). Whether these patients benefit most from aggressive PCI with long stents, surgical revascularization with CABG, or intensive OMT alone remains unknown [12,15,18,19,21,23,26].
- Technological refinement and accessibility: Continued research is needed to validate and refine non-hyperemic PPG, which would significantly streamline workflow and increase adoption [12,15,18,19,21,23,26,37]. Similarly, further validation of angiography-based PPG calculations (such as QFR-PPG) could make longitudinal physiological assessment possible without pressure wires, dramatically expanding accessibility [38,39,40].
- Integration with intravascular imaging: The relationship between PPG (a hemodynamic measure of pressure distribution) and underlying plaque morphology (burden and composition) remains incompletely characterized, with inconsistent findings across studies. Future studies should integrate PPG with intravascular imaging modalities such as intravascular ultrasound and optical coherence tomography. This would allow direct correlation between physiological patterns of pressure loss and underlying anatomical plaque morphology and composition [5,37,41]. Such approaches could identify high-risk features, such as thin-cap fibroatheromas, within hemodynamically significant focal lesions, potentially enabling truly comprehensive assessment of both physiological and anatomical vulnerability.

In conclusion, the PPG represents a fundamental advancement in invasive coronary physiology that has shifted clinical practice from evaluating individual stenoses to characterizing comprehensive disease patterns. This paradigm enables three critical improvements: (1) mechanistic resolution of FFR-iFR discordance, (2) prediction of functional outcomes after intervention, and (3) guidance for personalized revascularization strategies. Ultimately, PPG serves as an essential complement to FFR/iFR by shifting the clinical question from whether ischemia is present to how disease is distributed—a distinction that enables more precise, patient-centered interventions. Prospective randomized trials will solidify its role as a cornerstone of modern interventional cardiology practice.

## Figures and Tables

**Table 1:** Key Characteristics of Invasive Coronary Physiology Indices.

Index	What It Measures	Hyperemia Required?	Primary Output	Key Strength	Key Limitation
Angiography	Lumen diameter	No	Visual stenosis severity (%)	Anatomical roadmap	Poor correlation with ischemia
FFR	Maximal myocardial flow reduction	Yes	Pd/Pa ratio (≤0.80)	Gold standard for hemodynamic significance	Single-point measurement; does not assess disease distribution
iFR/RFR	Resting pressure gradient	No	Pd/Pa ratio (≤0.89)	Procedural simplicity; adenosine-free	Approximately 20% discordance with FFR
PPG	Longitudinal disease distribution	Yes (hyperemic) or No (resting)	Continuous index (0–1)	Quantifies focal vs. diffuse disease pattern	Requires pullback maneuver

FFR, fractional flow reserve; iFR, instantaneous wave-free ratio; Pa, aortic pressure; Pd, pressure distal to a stenosis; PPG, pullback pressure gradient.

**Table 2:** Summary of Selected Clinical Trials and Registries Evaluating PPG.

Study/Registry	Publication Year	Study Design	Patients/Vessels (N)	Key Question	Main Finding
Collet et al. (Validation)	2022 [7]	Prospective, multicenter	116 pullbacks (96 manual, 20 motorized)	Validation of manual versus motorized PPG	Excellent agreement and reproducibility of manual pullback PPG, enabling clinical application
PPG Global Registry	2024 [12]	Prospective, multicenter, international registry	993 patients (1044 vessels)	Predictive capacity for post-PCI FFR and impact on clinical decision-making	Excellent prediction of optimal post-PCI FFR (AUC 0.82); changed treatment strategy in 14% of patients; low PPG associated with higher periprocedural myocardial infarction
TARGET-FFR (Substudy)	2023 [19]	Randomized trial substudy	114 patients	Impact of disease pattern on physiology-guided incremental optimization strategy	Physiology-guided incremental optimization strategy applied more frequently in diffuse disease; however, focal disease patients achieved higher final post-PCI FFR, highlighting challenges of optimizing diffuse CAD

**Table 3:** Understanding FFR-iFR Discordance Through the Lens of PPG.

Discordance Pattern	Associated Disease Pattern	Typical PPG Value	Dominant Hemodynamic Force	Clinical Implication
FFR+/iFR−	Predominantly focal	High (median 0.82)	Separation loss (highly flow-dependent, $\;\propto v^{2}$)	Favorable PCI target with high potential for functional gain and symptom relief
FFR−/iFR+	Predominantly diffuse	Low (median 0.62)	Frictional loss (significant at rest, ∝ *v*·*L*)	PCI may yield suboptimal functional gain; consider higher procedural risk and potential for residual angina. OMT or CABG may be preferable

## Abbreviations

The following abbreviations are used in this manuscript:

AUC	Area under the curve
CABG	Coronary artery bypass grafting
CAD	Coronary artery disease
FFR	Fractional flow reserve
iFR	Instantaneous wave-free ratio
LAD	Left anterior descending
OMT	Optimal medical therapy
Pa	Aortic pressure
PCI	Percutaneous coronary intervention
Pd	Pressure distal to a stenosis
PPG	Pullback pressure gradient
